# Naming with Proper Names: The Left Temporal Pole Theory

**DOI:** 10.3233/BEN-2011-0338

**Published:** 2011-11-07

**Authors:** Carlo Semenza

**Affiliations:** Department of NeuroscienceUniversity of Padova and I.R.C.C.S. Ospedale S. CamilloLido di VeneziaItaly

**Keywords:** Proper names, left temporal pole, social cognition, anomia

## Abstract

Existing empirical data on proper names processing are critically reviewed in trying to understand which tasks may involve the left temporal pole, which proper name related functions are supported by this structure and eventually offer some speculations about why these functions might have developed in this location in the course of human evolution. While clinical group studies support the idea that proper name processing takes place in the left temporal pole, single case studies of selective proper name anomia or sparing, as well as neuroimaging studies, suggest the involvement of a larger neural network. Within this network, an important role may be played by the ventro-medial prefrontal cortex, including areas critical in social interaction. The differentiation in the brain of proper name processing from common names processing could in part be due to social pressure, favouring a neural system able to more efficiently and unambiguously sustain designating categories or designating individual entities. The activation of the left temporal pole in proper name processing is shown to increase with age. Longer social interaction may thus contribute to convey proper names processing toward areas closer to those supporting social cognition.

## 1. Introduction

Proper names have been shown to engage different functional processes with respect to common nouns. This difference was first suggested and then confirmed on the basis of several studies of neurological patients, where retrieval and memory for proper names were observed to be selectively damaged or selectively spared. A double dissociation was indeed found between common and proper names at different processing stages. Most studies were concerned with production, but in some cases the deficit also affected comprehension, including deficits of information stored in semantic memory. Thus while it was more commonly found on tasks involving lexical activation, a double dissociation was also found at the semantic level [1–9]. These findings favour the idea that processing proper and common names follows at least partially independent pathways in the cognitive system and in the brain. In fact these findings cannot be explained as resulting from a generic difficulty factor, especially if the two patients have been tested on exactly the same material, as it was done in at least one study [8], because what is difficult for one patient is shown to be easy for another patient and vice versa. This finding in itself does not however allow the inference that the two independent tasks are of equal difficulty. The problem of whether the independent pathways used to process proper and common names pose the same burden to the cognitive system is however a different problem, independent from their functional and anatomical separateness. One pathway, i.e. the proper name pathway, has been traditionally thought to be intrinsically more difficult to use. A full demonstration of the hardness of the proper name retrieval pathway has been however provided on neuropsychological grounds only in the past decade: the linking of proper names with their reference is more fragile than that of common nouns with their reference and may even require a larger amount of metabolic resources [7,9].

Neuropsychological findings, indeed, offered the first empirical support to theories, originated within philosophy and linguistics, positing fundamental differences in the functions of the two lexical categories [9–12]. The main theoretical idea is that, unlike com-

ISSN 0953-4180/11/$27.50 © 2011 – IOS Press and the authors. All rights reserved mon nouns, proper names possess *reference*, but not *sense,* i.e. they may be considered 'pure referring expressions', or, as some prefer, as locuses of identifying 'descriptions'. In short [9], it is sufficient to account for neuropsychological findings to think that proper names, as opposed to common nouns, denote individual entities but do not essentially rely on, or rely on only to a limited extent, sets of attributes. Thus, proper names essentially refer to individuals (or individual groups) while common names refer to categories. Another useful way of expressing this distinction is that proper names have only 'token' reference while common names carry 'type reference'. Neuropsychological findings offered important empirical support to these theories. In fact, proper name anomics were shown [2] to be unable to deal with other types of 'pure reference'. For example they are unable to perform efficiently in a paired associate learning test where the items do not have a semantic relation. This test requires, after hearing a few times a list of pairs of semantically related (North-South) or unrelated (pear-clock) words, the retrieval of one word of a pair given the other. Likewise proper name anomics cannot remember and never re-learn telephone numbers of people they knew before their illness, cannot retrieve the titles of popular pieces of classical music (indeed proper names) that they showed to know, or retrieve the dates of known famous events. The common denominator of these symptoms and proper name anomia is the inability to deal, at the retrieval level, with purely referential, non-descriptive, unique semantic relations. The main function lost by proper name anomics is thus believed to be that of retrieving the names of unique entities as opposed to retrieving the names of categories, labeled instead by common nouns.

The anatomical bases for the functional difference between proper names and common nouns is however far from being completely understood. In fact, localizing proper name production with respect to common nouns proves to be, notwithstanding clear neurological dissociations, a hard task. Findings obtained with the classic clinical-anatomical correlation method have been corroborated by lateralized teachistoscopic presentation, event related potentials and neuroimaging. The resulting general picture is not however unequivocal.

One very influential view, originally forwarded by Damasio, Grabowski, Tranel, Hichwa and Damasio [13], is that proper name processing takes essentially place in the left temporal pole (LTP). Such view is not however without problems. While surely the LTP is likely to play an important role, empirical evidence accumulated over the past years suggests that proper name processing is not confined to this brain location. It is still unclear, moreover, what exactly the LTP might do. It is the aim of the present review to discuss existing empirical data in trying to understand which tasks may really involve the LTP, which functions are supported by this structure and eventually offer some speculations about why these functions might have developed in this location in the course of human evolution.

## 2. Empirical findings originating the left temporal pole theory: clinical group studies and the functional imaging approach

The LTP theory, in short, states that the LTP is crucial for retrieving words for unique concrete entities. A throughout review of empirical data claimed to support this view can be found in Tranel [14]. These data stem from a number of clinical group studies, paralleled by neuroimaging investigations.

### *2.1. Clinical group studies*

The initial clinical study pointing out the role of the LTP in proper naming is that of Damasio et al. [13]. It was conducted in a large group of neurological patients with focal brain lesions in a chronic phase, and their naming abilities were measured with a detailed series of standardized tests. These patients did not have general intellectual impairment, understood the task and did not have problems in attending to or perceiving visual stimuli. They were administered with pictures of famous persons, presented as faces, and pictures of animals and tools. The naming score was calculated by dividing the number of items named correctly by the number recognized correctly (and converting to percent). Naming scores that were two or more standard deviations below the mean of the normal comparison group were considered as pathological. 13 patients out of 127 were thus found to have impaired person naming. Looking at their lesions, the highest region of overlap was indeed the LTP, including both cortex and subcortical white matter. Importantly, in 7 out of these 13 patients the deficit was restricted to the category of persons, while their performance with animals or tools was instead within two standard deviations from the normal mean.

A replication and extension of this study was conducted by Damasio, Tranel, Grabowski, Adolphs and

Damasio [15]. The proper naming test and scoring procedures were the same as in the initial study. 139 patients, including those reported in the earlier study, were included in the investigation. This time, instead of calculating an overlap map for the patients with impaired naming, two overlap maps were calculated, one for impaired patients and one for unimpaired patients. The unimpaired map was then subtracted from the impaired map. Areas specifically related to impaired retrieval of proper names were considered those where, at a given voxel, there were at least five more impaired patients relative to unimpaired patients. 39/139 patients had proper naming scores two standard deviations below the normal mean. A marked concentration of impaired>unimpaired patients was found in the LTP. There was no other brain region associated with such a concentration of impaired cases. A small proportion of patients with problems in proper naming was however found with lesions outside the LTP.

An addition to these studies has been later provided by Tranel [16]. 11 patients with a lesion in the LTP were compared to 10 patients with a right temporal pole (RTP) lesion and 90 healthy control participants. The LTP group's mean score on person naming was significantly lower than that of the other groups. In the same article Tranel [16] reports another study, on 74 patients, focussing on naming of famous landmarks. The scoring system was similar to the other studies. The LTP group (11 participants) scored on average significantly lower than patients with an RTP lesion as well as patients with lesions that did not affect the temporal poles.

### 2.2. *Neuroimaging*

Clinical investigations described in the preceding session [13,15,16] were paralleled by neuroimaging studies using PET. Damasio et al. [13] compared naming of familiar faces to a baseline task requiring participants to judge the orientation of unfamiliar faces presented either upright or upside down and respond "up" or down". Two main areas of significant activation distinguished proper naming from the baseline task: the LTP and the RTP. Thus, while activation of the LTP confirmed the results of the clinical investigation, activation of the RTP was interpreted as reflecting the face recognition process. A first follow up study, conducted with a similar methodology [17] obtained exactly the same results as far as naming faces was concerned. This study showed the same results also when naming was required of pictures of famous landmarks. This finding was interpreted as evidence that the left temporal pole is critical for retrieving the names of unique entities such as persons and landmarks. A further PET investigation by Damasio et al. [16], however, yielded similar but indeed more complex results. In this study the areas of activation in naming faces (as compared to animals and tools) were: (a) the RTP; (b) the left anterior sector of superior temporal sulcus; (c) the ventromedial prefrontal cortices; and (d) anterior cingulate. Regions related to the processing of emotions (i.e. the ventromedial prefrontal cortices and anterior cingulate cortex) would be required because unique familiar faces, for most subjects, are emotionally salient stimuli. Remarkably, the activation of the LTP only reached a marginal level of significance. As Damasio et al. [16] recognized, this PET study was not designed to separate processes involved in concept and name retrieval.

Further indications of an activation of the LTP in proper name retrieval (as well as other tasks concerned with proper names, e.g. choosing at multiple choice the proper name associated with a face), although most often in connection with other areas, come from fMRI studies [18–23]. Relative to other brain regions, however, fMRI image quality may suffer from signal deficits in comparison to PET [14]. Most of these studies were however able to confirm the finding that the LTP activates in the retrieval of face-name associations. The most original contribution of fMRI studies has been to show that, while the left anterior temporal region is crucial for the retrieval of people's names irrespective of their familiarity, the right superior temporal and bilateral prefrontal areas are crucial for the process of associating newly learned people's faces and names [21]. The relevance of these findings will be fully discussed later in this paper.

## 3. The clinical-anatomical correlation: single case studies

Reviews of single case studies of selective proper name anomia seem to tell, compared to group studies, a more complicated story. Lesions to the left hemisphere found to cause selective proper name anomia, in fact, are manifold. They encompass the left basal ganglia (including amygdala), the left thalamus, the temporal lobe, and the postero-temporal/occipital lobe. Reviews of available cases of discrepancy in the production of proper and common names [7,24,25] allow indeed the following conclusions: (a) in proper name anomia the left temporal lobe is often damaged; (b) several proper name anomia cases are determined by lesions clearly outside the left temporal lobe (e.g., patient PC, [1]); (c) the left temporal lobe is most often damaged in selective sparing of proper names; (d) at least two cases of selective sparing of proper names follow a lesion in the LTP (e.g., patient ACB [8]).

The best evidence against the role of the LTP in naming comes from two cases reported by Pavão Martins and Farrajota [8]. Contradicting its crucial role in proper name processing, the LTP was damaged in ACB, a patient with sparing of proper names, but undamaged in JFJ, who suffered proper name anomia! These two patients had a comparable age and education and came from the same social milieu. Importantly, they were tested using the same material, thus this double dissociation cannot result from any unbalance between the tests tapping each of the two name categories.

While in all these studies, as in group studies, the main task was always picture naming, most of the patients show anomia for proper names also in other tasks, like naming on definition or naming by category (e.g. politicians, musicians, etc.). Moreover, while most patients were not sensitive to phonemic cueing, patients were also found who showed significant improvements on a cued condition [26].

## 4. Problems with the LTP theory

Single case studies, on the whole, do not seem to unambiguously support the LTP theory. Damasio et al. [15] express the view that cases contradicting the LTP theory should be considered as exceptions. Such cases were indeed found also in their sample, although their frequency was rather low. Fully studied single cases whereby the lesion leading to proper name anomia or proper name sparing is inconsistent with such theory are however not so infrequent and must be taken seriously. It is important to underline the relevance of these findings.

Unlike in the case of group studies, in each single case the dissociation between proper names and common nouns is clearly demonstrated in a series of tasks, in particular those tapping the lexical retrieval level. Such dissociation is not guaranteed in the group study approach. A full criticism of the original Damasio's et al. [13] methodology is first found in Caramazza and Shelton [27], and similar arguments have later been provided by Semenza [7,9]. Indeed patients who retrieve proper names with an efficiency lower than two standard deviations from the norm, the selection criterion used in Damasio et al. [13], may still have no significant difference between proper name and common name retrieval. The more sophisticated distribution analysis approach used in Damasio et al. [15], meant to pre-empt the criticism that the classification into normal/abnormal categories based on a two standard deviations difference from the mean of normal controls provides an arbitrary dichotomy, does not seem to fully answer the main problem. In fact, in the distribution analysis, the comparison was just made between pathological participants and controls. A crucial comparison that needs to be done is however the comparison in each participant between the performance on proper names and the performance on common nouns. Otherwise a given patient may still be classified as proper name anomic while in fact his/her performance with proper names does not differ from that on common nouns.

Neuroimaging studies are not without problems for the LTP theory either, despite some converging evidence in its favour. As previously mentioned, Damasio et al. [15] showed how other left hemisphere structures like the ventromedial prefrontal cortex, the anterior cingulate, the anterior sector of superior temporal pole as well as the right temporal pole were all activated in naming person faces, while activation in the LTP only reached a marginal level of significance. Damasio's et al. [15] results confirm on the whole those reported by Proverbio, Lilli, Semenza and Zani [28] in the only ERP study carried out so far on the subject. Participants in this experiment, after looking at short definitions (e.g. 'the city where FIAT headquarters are'), were presented with a trigram (e.g. 'RIN'), and they had to decide whether this trigram appeared in the name ('Torino') corresponding to the definition. This tacit retrieval of a proper name's phonological form elicited a strong activation of left anterior temporal and left centro-frontal areas, while the same task shows greater involvement of occipito-temporal areas with common names. Together with Damasio et al. [15], this study thus emphasizes the contribution of left frontal areas in proper naming. Recent support for the contribution of left frontal areas to proper naming comes also from a study conducted via direct cortical stimulation [29]. However in this study face-naming specific sites were found to be more extended with respect to those shown by neuroimaging, including the superior, medial and inferior frontal lobe gyri, as well as the anterior part of the superior and medial temporal gyri. Surgical removal of the frontal part of the left uncinate fasciculus, the structure connecting the LTP to the orbito-frontal cortex results in selective difficulties in naming people [30]. The co-working of the LTP and frontal areas in processing proper names seems just confirmed, although not yet with the desirable precision. Surely anomia for proper names can occur without apparent frontal lobe damage. Thus the contribution of the frontal lobe to proper name processing may be limited to specific tasks and conditions that must be better specified.

A further problem with studies supporting the LTP theory is that they are limited to confrontation naming (of faces or pictures of places). Indeed the best data in favour of the LTP theory come only from group studies in face naming but do not emerge as clearly when proper names were prompted by definitions. For example [31], patients with left and right temporal lobe epilepsy were compared on both picture naming and definitional naming of famous people. Left temporal lobe epileptics were worse than right temporal lobe epileptics on naming pictures, but no difference emerged in naming on definition. It is important to stress the fact that naming faces may dissociate from producing proper names on definition, as clearly demonstrated by the "prosopanomia" case described by Semenza, Sartori and D'Andrea [32]. The patient, VI, who was not prosopagnosic, suffered from a face-specific form of optic aphasia. He had no trouble in identifying the face (given the name, he could point to the face on multiple choice), but, like optic aphasics, he was unable to retrieve enough information from the visual stimulus (in his case only faces) to activate the name. In contrast, he was good at naming people on definition and when shown a picture of a person, he always provided correct semantic information about each known individual he could not name. Semenza et al. [32] demonstrated that VI's problem specifically concerned naming faces, and did not consist of the inability to retrieve any proper name upon picture presentation. The patient was in fact fairly good with geographical items in a map and could name on confrontation individual car models (e.g. Corvette) or the makers of Venetian vases. He suffered of a very small surgical lesion, resulting from the removal of a colloid cyst, immediately anterior to the third ventricle.

In conclusion, the main problems limiting the LTP theory can be summarized as follows: a) the LTP theory is not altogether sustained by single case reports, while group studies may have methodological shortcomings; b) other anatomical structures, including left frontal areas, as suggested by neuroimaging and electrophysiological investigations seem to be involved in proper name retrieval; c) the only task whereby the LTP theory might apply to a certain extent is picture naming; all other tasks related to proper naming have not been studied enough. Indeed, as Tranel [16] argues, 'depending on factors such as the exact nature of the stimulus, task demands, and the nature of a particular individual's abilities, the same task might engage different convergence zones across relatively comparable individuals'.

## 5. What may the LTP do with proper names?

Studies supporting the LTP theory, despite their problems, must capture some truth. A number of recent investigations may help in understanding the role of the LTP, its relation with other structures in proper name related tasks, and why this area may have developed in evolution to accomplish proper naming. There are still unsolved issues, mainly concerning the nature of each different proper name related task. Indeed little effort has been made to distinguish and experimentally contrast different tasks. Thus only very general conclusions can be drawn at the present time and the issue of localization of each specific proper name related task remains unsolved. Nonetheless recent contributions may represent a significant step forward in several respects.

An important series of fMRI experiments [20,21,33] investigated, in the past decade, the association of people names with faces and person specific semantic information. According to these studies [33] the LTP may mediate associations between names and person-related semantic information, whereas the right temporal pole (RTP) mediates the association between faces and person-related semantic information in memory for person identity information. In addition, the RTP may contribute to a more general relational processing of associative components, including memory for person identity information. The LTP may thus be involved in proper name related processing other than retrieval. Both the LTP and the RTP would contribute to the retrieval of newly learned people's names [21].

Other, very interesting, recent contributions concern two previously neglected factors that may have shaped the LTP functions: social interaction and age. One influential idea is that the LTP plays a special role in semantics. According to Patterson, Nestor and Rogers [34], on the basis of evidence from patients affected by semantic dementia, the left anterior temporal lobe would indeed serve as a hub binding all semantic attributes together. Others [14,35] suggest, in contrast, that such hub role is specifically played there only for the knowledge of unique entities, such as those labelled by proper names. While it is not the aim of the present paper to discuss the merits of the hub theory, such theory is worth mentioning because some aspects of the discussion it stimulated may be relevant here.

In discussing the hub theory, Simmons and coworkers [36,37] suggested that the LTP is specifically involved in supporting socially relevant conceptual knowledge. These authors found person-selective but not domain-general responses in both the LTP and the RTP when subjects encoded semantic facts. They also stress the point that these structures are engaged in social conceptual processing (see also, for instance [38]). The temporal poles would thus work in unison with a wider social cognition system to support learning facts about others.

As Simmons and Martin [36] argue, the fact that the LTP is involved in processing social conceptual knowledge generally may be accommodated with the fact that it is also implicated in processing information about unique entities. Information about faces, especially, and, to a lesser extent, landmarks, is important for social interaction. In this respect, Simmons and Martin [36] observe that while anomia for people names is observed either in isolation [39] or together with anomia for geographical items, there are no descriptions so far of an isolated deficit for landmarks (see [9] for a discussion on this issue).

The social aspect of proper name processing may also interact with age, a factor that has been long known to affect proper naming [40,41]. For example, Yucus and Tranel [42] showed that preservation of proper names following left temporal lobectomy is associated with early age of seizure onset in temporal lobe epilepsy. More recently differences were investigated via fMRI in brain activations between healthy young and older adults during the successful retrieval of people's names and job titles associated with faces [43]. The hippocampus showed significant retrieval success activity in both tasks and the activity was greater for young compared to older subjects. The LTP showed greater activity for names of faces than for job titles, but there was no age difference in the activity in this region. Finally, functional connectivity between hippocampal and LTP activities in both retrieval tasks was higher for young than for older adults. The same issue has been very recently directly addressed in a different experiment by Ischebeck, Steindl, Reishofer, Koschutnig, Semenza, Ebner and Neuper [44]. These authors also showed activation of the LTP in encoding proper names: however this activation looked significantly stronger in older subjects than in a younger group. Longer social interaction apparently may thus contribute to convey proper names processing toward areas closer to those supporting social cognition. In other words, consistently with Simmons and Martin's [36] view, the LTP would accumulate both social conceptual knowledge and (indeed important for social interaction) information about unique entities acquired in life. Its close and privileged connections with the frontal lobe [30], and with the wider social cognition system, would thus be increasingly reinforced. Stronger activation of LTP in the elderly would thus reflect relying on this system in order to overcome the higher demands of proper name processing relative to processing of common nouns.

The differentiation in the brain of proper name processing from common names processing, indeed, may have probably found its origin in social pressure. The main difference between the two categories lies, as reported in the introduction, in the contrast between the function of categorization and the function of individual designation. As Semenza [7,9] argued, in the primitive world it might have become increasingly useful not only to be able to categorize surrounding entities but to label such categories in a precise fashion. Using a precise name, e.g. 'lions' or 'swamps', respectively, for a kind of big wild cat and for a kind of wet dangerous place, might have, for example, saved con-specifics' lives by allowing quicker and more efficient descriptions and warning. Likewise, the ability to single out and call by a proper name individual entities ('X' is good, 'Y' is bad; 'Z' is a dangerous place) might have served similar warning aims. In the particular case of naming fellow humans, additional social purposes, also useful for reproduction, might have determined an advantage for the individual gifted in this respect. Thus, those humans with a neural system that more efficiently and unambiguously sustained designating categories or designating individual entities (distinct functions may allow quicker and more precise processing) might have had a greater chance of survival and of mating, and in this way their neural systems would have been winners in natural selection. That's why in humans' evolving brain the increasingly important semantic function of individual designation might have moved forward, in closer connection to frontal lobe areas where social action was supported.
